# From waste to health: exploring the male sexuality enhancing activities of the bioactive components of orange peel

**DOI:** 10.3389/fendo.2025.1598545

**Published:** 2025-07-11

**Authors:** Adeyemi Fatai Odetayo, Grace Edet Bassey, Adenike Lizzy Adeyemi, Luqman Aribidesi Olayaki

**Affiliations:** ^1^ Department of Physiology, Faculty of Basic Medical Sciences, Federal University of Health Sciences, Ila-Orangun, Nigeria; ^2^ Department of Medical Physiology, Faculty of Basic Medical Sciences, University of Uyo, Uyo, Nigeria; ^3^ Environmental Health and Safety, Osun State Ministry of Health, Osogbo, Nigeria; ^4^ Department of Physiology, University of Ilorin, Ilorin, Nigeria

**Keywords:** antioxidant, citrus peel, erectile dysfunction, flavonoids, phenolic compounds, traditional medicine

## Abstract

Large amounts of citrus wastes, especially orange peels, are produced globally, and they are an environmental menace in different parts of the world because of their perishability. However, various research findings have shown that orange peels are a rich source of vitamins, fibers, phenols, and other bioactive compounds. This makes orange peel a promising therapeutic option for managing various disease conditions. Despite these health benefits, only about 10% of orange peel produced as waste is recycled and reused. Hence, the review was designed to explore the beneficial roles of orange peel bioactive compounds on male sexual function. This review, therefore, explore the aphrodisiac effect of orange peel bioactive compounds by reviewing recent publications that detail the association of orange peel bioactive compounds with male sexual functions regarding its potential roles in erectile function regulation, testosterone levels, and semen characteristics. Bioactive compounds from orange peel contributed positively to sexual and erectile functions. This was mediated via a hormonal-dependent mechanism and upregulation of steroidogenic enzymatic activities. It also improves endothelial functions by stimulating the secretion and release of nitric oxide, which is important for erection. Furthermore, it acts by mediating the activities of monoamine neurotransmitters, which are responsible for the motor activities involved in sexual function. Orange peel bioactive compounds enhance male sexual functions via multiple pathways. Hence, orange peels offer great potential for developing functional food products with health and economic benefits.

## Graphical Abstract

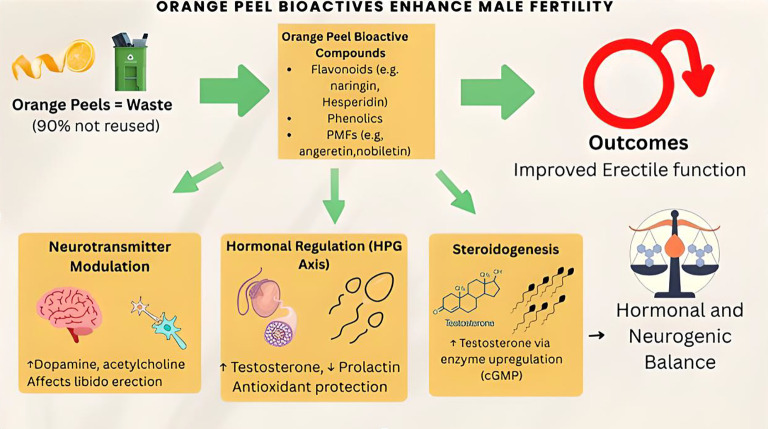

## 1 Introduction

**Figure 1:**
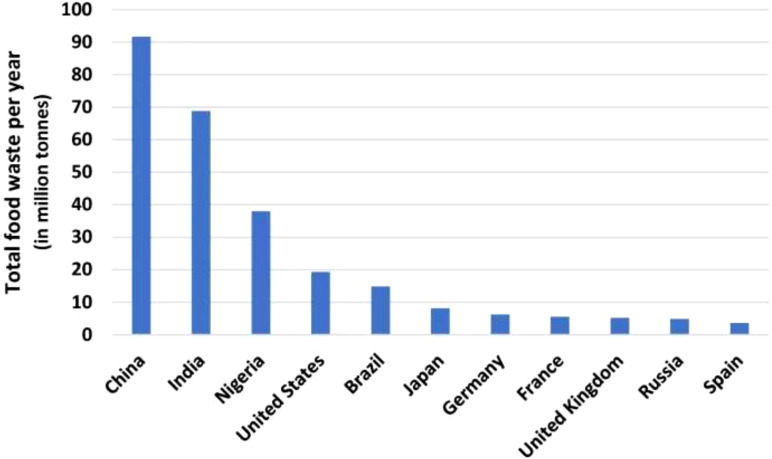
The top eleven countries with the highest annual food waste (4).

The current mounting rate of environmental pollution has significantly raised different researchers’ interest in the efficient use and recycling of agricultural residues and waste. Agricultural activities account for a considerable amount of waste annually, which, if not effectively managed, can result in socio-economic, environmental, and health problems (1). It was reported that about one-third of the food produced for human consumption is lost or wasted annually (2). The estimated 931 million tonnes of food waste generated yearly, of which 61% comes from households, 26% from food services (restaurants), and 13% from retail, make up 17% of the world’s total waste (3). As shown in **Figure 1**, the annual food waste in millions of tonnes for the top ten nations worldwide revealed that the China, India, Nigeria, and United States produce the most food waste annually, with 91,646,213 tonnes, 68,760,163 tonnes, 37,900,000 tonnes, and 19,359,951 tonnes, respectively (4). The residues (such as peels and seeds) arising during household consumption and industrial processing of different agricultural products, such as vegetables and fruits, result in huge waste (5).

Recently, the food processing industry has rapidly grown and, at the same time, has significantly increased food waste globally (6), valued at around $1 trillion, with the US singlehandedly generating 60 million metric tonnes per year (3). Regrettably, waste generated from fruits such as peels, which are rich in beneficial compounds, contributes significantly to this waste. These agricultural wastes are mainly disposed of in rivers, landfills, and open dumps, leading to air, soil, and water pollution. Unfortunately, despite the nutritional value of this generated waste, only about 10% of the waste is recycled and reused. This contributes significantly to the increased rate of economic losses and environmental pollution.

Based on the above, it is important to establish effective strategies for the optimal utilization of the generated agricultural waste. Organic residues contain different bioactive molecules, such as phenolic compounds, which are important for various biological activities responsible for human health (7). The extraction, identification, and investigation of these bioactive molecules will contribute significantly to the use of agricultural waste and prevent economic losses and environmental pollution.

Among these agro-based waste products, orange peel is gradually becoming the most researched waste product owing to its rich phytochemical profile (8). The primary orange peel-producing countries are Brazil, China, India, the USA, and Mexico (9). Within Europe, Spain, Italy, Greece, and Turkey are major orange producers and waste generators, particularly in the Mediterranean region. Other countries that generate large amount of orange waste include Egypt and South Africa (10).These nations collectively account for a significant portion of global orange production, leading to large volumes of orange peel generated during processing and consumption. The generated orange peels are considered as waste and have been identified as the primary substance disrupting the cleanliness of urban areas. Orange peel is a by-product obtained after juice extraction or other orange processing activities. This by-product is perishable, and proper disposal is a significant challenge faced by pollution monitoring agencies and processing industries (11). Hence, there is a need to utilize orange peel to produce value-added food products. Using orange peels will provide health benefits and resolve environmental pollution caused by orange peel perishability. Interestingly, some countries consider orange peel a perfect sweet and chewy snack, especially when consumed with dark chocolate (6). Also, scientists are exploring the rich phytochemistry of this agricultural waste to develop functional foods and dietary supplements. The antibacterial property of orange peel has made it a natural preservative that could replace artificial ones in different food products (12). In fact, orange peel has been proven to be effective for managing complications arising from disorders such as cardiovascular dysfunction (13), diabetes (14), gonadotoxicity (15) and cancer (16). Importantly, the exploitation of orange peel in food will not only lead to the cost-effective, innovative therapeutic option, it also improves the value of functional and nutraceutical food products (17). Aside from the health benefits, the recovery of the orange peel will also reduce the global waste burden. Orange peel has been valorized for its phenolic content, e.g., phenolic acid and flavonoids. It has been established that phenolic compounds of natural origin are more desired due to their potent antioxidant properties in food systems or living organisms for various maladies (12). Hence, this review article aimed at establishing the aphrodisiac and fertility-enhancing properties of orange peel bioactive compounds.

## 2 Orange peel: an insight

Orange is the world’s most popular fruit and belongs to the Citrus genus. It is the largest citrus producer, accounting for more than half of all the citrus fruits produced globally. Orange can be classified into sweet orange (Citrus sinensis), sour or bitter orange (Citrus aurantium), mandarin orange and tangerine varieties, Citrus reticulate, and Hybrid oranges. Sweet oranges are the most important in production volumes and cultivation areas (18). In most markets, juice made from sweet oranges is regarded as orange juice.

An orange is a ball-like fruit protected by waxy skin called the peel. The orange peel comprises a thin outer layer known as the flavedo and a thicker inner layer referred to as the albedo (19). The flavedo consists of the carotenoids that account for the fruit’s characteristic color (20), and vesicles (small sacs/cavities) that contain the peel oil. This peel oil accounts for the fruit’s fresh aroma. On the other hand, the white spongy inner albedo is made up of different substances such as flavonoids, d-limolene, limon, and pectin (21).

As shown in **Table 1**, orange peels are excellent sources of bioactive compounds such as essential oils, polyphenols, fibers, minerals, pectin, and monosaccharides (22). The essential oil contains different terpenes, with limonene as the primary compound. It also contains oxygenated compounds such as alcohols, aldehydes, and esters (23). Polyphenolic compounds are other bioactive substances in the form of phenolic acids, flavonoids, and their derivatives (23, 24). Additionally, orange peels are rich in sugars, which can be as free monosaccharides, disaccharides (sucrose), or polymerized to cellulose (glucose), hemicellulose, and pectin. Pectin is a heteropolysaccharide rich in galacturonic acid (22).

**Table 1:** Chemical composition of orange peel.

Components	Chemical Composition
Total sugar content	165 mg/g
Pectin	128 mg/g
Crude fiber	86 mg/g
Crude protein	42 mg/g
Lignin	22 mg/g
Total ash	21 mg/g
Crude Fat	15 mg/g
Carbohydrate	715.7 mg/g
Phenolic compounds	179 mg/g
Vitamin C	65 mg/g
B-carotene on dry weight basis	0.021 mg/g
Hesperidin	0.066-66 mg/g
Narirutin	0.03-26.5 mg/g

12.

Of these bioactive compounds, the most important are the flavonoids, and the most prevalent are hesperidin, naringin, and rutinose (25). Hesperidin is abundantly found all over the peel, whereas naringin is predominant in the flavedo portion (26). Other important flavonoids include neohesperidin, didymin, narirutin, eriocitrin, and tangeretin. Another important bioactive substance is polymethoxylated flavone (PMF), a mixture of 5.44% hydroxylated PMFs and 75.1% non-hydroxylated (27). PMFs such as nobiletin, nestin, and tangerine are majorly present in the flavedo portion of the essential oil, but they are less frequent than flavanones. PMFs are commonly present in sweet and bitter oranges, such as nobiletin and tangeretin (28).

## 3 Orange peel bioactive components extraction methods

Orange peel extraction has been a focal point for developing a valuable strategy to exploit waste material from the orange juice industry (29). These methods are designed to optimize bioactive compound yields and quality, including essential oils, pectin, and d-limonene, and contribute to environmental sustainability. Several sophisticated techniques have been developed with unique advantages, drawbacks, and utility.

### 3.1 Supercritical fluid extraction

Supercritical fluid extraction (SFE) represents one of the greenest extraction methods. It employs supercritical carbon dioxide (ScCO_2_), which is frequently supplemented with ethanol, as an organic solvent of choice (30). This approach is based on controlled temperature and pressure conditions and thus delivers good quality products with a low environmental footprint. Ghadiri et al. (31) demonstrated that optimizing SFE parameters such as temperature, pressure, and extraction time yields essential oils with superior quality while reducing energy consumption and solvent waste. Similarly, Ortiz-Sanchez et al. (32) reported that SFE outperformed conventional methods in selectively isolating bioactive molecules like hesperidin, making it a preferred choice for high-value products.

This process requires a pump for CO_2_, a pressure cell that will contain the sample (orange peel), a restrictor to regulate control flow and pressure of the extraction fluid. The extracted fluid is pumped to a heating zone to ensure it is heated to supercritical conditions. This fluid will then move to the extraction vessel where it is immediately diffused into the solid matrix where the materials to be extracted will be dissolved. The dissolved material is then swept into a separator from the extraction cell where it will settle out. The CO_2_ can then be cooled, re-compressed, or discharged into the atmosphere.

The major advantage of this method is it selectivity and efficiency in extracting specific compounds from mixtures, while also minimizing the use of organic solvents and energy consumption. However, it requires high operational cost, which can be a barrier to widespread adoption, particularly in specialized fields.

### 3.2 Microwave-assisted extraction

Microwave-assisted extraction (MAE) has become a trend in terms of energy efficiency and shortened extraction time. One major advantage of this method is that it is rapid since the peel will quickly heats and the matrix of orange peel will quickly breaks down, liberating bioactive compounds more efficiently. The microwave extraction method requires heating the orange peel via the use of radiation energy that has short wavelength and high frequency. The water present in the peel absorb the microwave’s energy to increase the internal temperature. There is a rapid increase in temperature of the vascular bundle and glandular cell system and this temperature can be maintained until the internal pressure exceeds the capacity of the cell wall to expand, causing the cell to rupture and the spices located inside the cell to flow out of the cell wall, transfer to the surrounding extraction medium. Benassi et al. (33) showed that MAE yielded higher values of pectin yields at lower energy expenditure compared to other strategies, including hot-water extraction and Rapid Solid-Liquid Dynamic (RSLD) extraction. Furthermore, Murador et al. (34) investigated the potential of ionic liquids based on MAEs to improve the bioaccessibility of carotenoids and chlorophylls and thus showcased the potentially broad applicability for nutraceutical purposes.

The advantages of this method of extracting bioactive compounds from orange peel include its fast extraction times and higher yields, along with reduced solvent consumption. However, there is possibility of analyte degradation and the need for specialized equipment that are quite expensive.

### 3.3 Ultrasound-assisted extraction

Ultrasound-assisted extraction (UAE) is a non-thermal extraction method that uses sound waves to enhance the extraction of bioactive compounds from plant materials. It leverages acoustic cavitation, where the high-frequency sound waves create tiny bubbles that implode, generating localized heat and pressure. This process disrupts cell walls, facilitating the release of desired compounds into the solvent. This approach is particularly useful due to its small solvent uptake and ability to retain heat-sensitive molecules. Li et al. (35) have shown the role of UAE in obtaining phenolics and pectin from orange waste with the yield of high-quality extract for nutraceutical and pharmaceutical purposes. UAE’s low power demand and operational ease confer practicality for industrial-scale development.

### 3.4 Hot-water extraction

Although hot-water extraction is one of the oldest methods, it continues to be extensively employed, especially in pectin extraction. It is based on using water as a solvent at specific temperatures and pH conditions to perform the reactions in high yields with a low environmental footprint. Benassi et al. (33) also pointed out the ability of this solution, emphasizing its applicability for industrial-scale applications where simplicity and low cost are necessary.

### 3.5 Solvent-based extraction

Although less environmentally acceptable than green options, solvent-extractable-based extraction remains valuable in specific applications. Siddiqui et al. (36) highlighted bio-solvents’ advantages, including their potential to replace expendable heptane and, consequently, the environmental impact. This method is still feasible for compound mining, such as d-limonene, especially after the optimization of efficiency and safety.

## 4 Male sexual function

Sexual function is the ability to experience sexual pleasure or react sexually and is characterized by the ability to move through the stages of sexual desire, arousal, and orgasm without difficulty. Male sexual function is regulated by a complex interplay between the psychological and physiological mechanisms that significantly impact the overall life quality (37). This mechanism involves the activities of the neurological, cardiovascular, and endocrine systems. Hence, any alterations in these systems can impair male sexual function, leading to sexual dysfunction. Therefore, sexual dysfunction is not a singular disorder but a multifaceted issue that disrupts life quality (38). One of the most prevalent sexual dysfunction is erectile dysfunction, an inability of the penis to achieve and sustain erection for a satisfactory sexual experience (39, 40). Hence, penile erection plays a dominant role in maintaining sexual function.

Penile erection is a highly intricate process requiring the congenial relationship between the psychological, endocrine, neurological, and cardiovascular aspects of the body systems (40). Neuromuscular activities play a central role in penile erection mechanisms, and they involve an interplay between the hormones, neurotransmitters, autonomic nervous system, and nitric oxide (NO). Once there is sexual desire or arousal, the nervous system will stimulate the release of NO (a potent vasodilator) via dopamine-oxytocin-NO signaling (41, 42), leading to the relaxation of the smooth muscles present in the corpus cavernosal via the NO- cyclic guanosine monophosphate (cGMP) dependent pathway (43, 44). Consequently, an increase in blood flow to the corpus cavernosum and a decrease in local venous return occurs, leading to penile erection.

### 4.1 Neurobiology of male sexual function and orange peel bioactive compounds

#### 4.1.1 Erectogenic enzymes and orange peel bioactive compounds

Sexual desire, also referred to as libido, is the first stage of the sexual cycle, and it can be defined as the drive to engage in sexual activity or the interest in sexual objects and activities. On the other hand, sexual arousal is a psychological and physiological response that occurs as a result of exposure to sexual stimuli or preparation for sexual intercourse (penile erection) (45). The mechanisms underlying sexual arousal are complex and involve different cerebral circuits involved in the release of neurochemicals and sex hormones. The ascending pathways consist of five neurochemical systems: dopamine, serotonin, acetylcholine, norepinephrine, and histamine. These neurochemicals are majorly involved in the arousal of the forebrain. (46).

Dopamine is a monoamine neurotransmitter that facilitates sexual motivation and penile erection via its action on the oxytocinergic neurons that are present in the hypothalamic paraventricular nucleus (PCN) and the pro-erectile sacral parasympathetic nucleus of the spinal cord (42, 47). Also, serotonin is another monoamine neurotransmitter that is responsible for maintaining sexual activities. Unlike dopamine, serotonin inhibits sexual function (48). In fact, the selective serotonin reuptake inhibitor class of antidepressants has been shown to impair penile erection and sexual desire (49, 50). Another important monoamine neurotransmitter involved in sexual function is acetylcholine. Acetylcholine has been shown to have an excitatory effect on penile erection by stimulating the relaxation of the cavernosal smooth muscles (51). The presence of cholinergic nerves and receptors in the corpus cavernosum (46, 52) further substantiates the important roles of acetylcholine in penile erection.

Additionally, acetylcholine stimulates the release of dopamine responsible for sexual motor activities (53). Norepinephrine or noradrenaline is also a monoamine neurotransmitter, and it can also be considered a stress hormone. The noradrenergic neurons in the brain are part of the neurochemical system with excitatory effects on mental alertness, sexual arousal, and the reward system (46). Histamine is also a neurotransmitter that stimulates the hypothalamic ventromedial nucleus, leading to the modulation of sexual behavior (54). In fact, histamine antagonists have been implicated in loss of libido and erectile dysfunction (55). In sum, monoamine neurotransmitters play a dominant role in sexual arousal.

Orange peel fertility-enhancing abilities can be associated with their modulatory effects on monoamine neurotransmitter activities. Orange peel flavonoids such as heptamethoxyflavone have been shown to increase the hippocampal brain-derived neurotrophic factor (BDNF) expression (56) via extracellular signal-regulated kinases 1/2 (ERK1/2) and cAMP response element-binding protein (CREB) signaling (57). In the same vein, Sawamoto et al. (58) revealed that heptamethoxyflavone stimulated BDNF synthesis and release in the C6 glioma cells. Another important orange peel flavonoid that plays a key role in sexual function via BDNF synthesis is hesperidin. Hesperidin is an antidepressant, and its antidepressive activities have been shown to increase the production of BDNF in different animal behavioral tests (59, 60). This BDNF synthesis has a role to play in penile erection, and together with its receptors, BDNF is a key regulatory protein in the male genitourinary system and can be a therapeutic target for the management of secondary penile dysfunction (56). BDNF exhibits this fertility-enhancing capacity by modulating the activities of monoamine neurotransmitters (61–63). This was also confirmed by the study of Juric et al. (64), which established a positive relationship between BDNF concentration and monoaminergic system activities.

Additionally, naringenin, a dietary flavonoid present in orange peel, has been shown to increase the activities of the monoamine neurotransmitters by acting as a monoamine oxidase inhibitor (65). Monoamine oxidase inhibitors are chemicals that break down monoamine oxidases, enzymes that breakdown monoamine neurotransmitters in the brain. Hence, via its monoamine oxidase inhibitor activities, naringenin increases the level of monoamine neurotransmitters in the brain, thereby increasing sexual function. Therefore, one of the mechanisms of action associated with the fertility-enhancing properties of orange peel bioactive compounds is via their modulatory effects on monoamine neurotransmitter activities.

#### 4.1.2 Reproductive hormones and Orange peel bioactive compounds

Male reproductive hormone concentration is majorly regulated by the hypothalamus via the hypothalamic-pituitary-gonadal (HPG) axis. HPG axis is a closed-loop relationship between the hypothalamus, pituitary, and testis. The hypothalamus synthesizes gonadotropin-releasing hormone (GnRH), which acts on the pituitary gland to produce luteinizing hormone (LH) and follicle-stimulating hormone (FSH). These two hormones act on the testis, but while LH stimulates the Leydig cells to produce testosterone (a major sex hormone in males), FSH stimulates spermatogenesis via its stimulatory effect on the Sertoli cells. The produced testosterone sends negative feedback to the pituitary and/or hypothalamus to inhibit their secretions. Also, the synthesized testosterone can be converted via aromatization in the brain to produce estrogen. Estrogen and testosterone are important for neural circuit development, sex-specific behaviors, and orientation (46). The activities of this closed-loop relationship can be disrupted by the hypothalamic-pituitary-adrenal (HPA) axis which becomes activated during stressful conditions. This HPA axis inhibit the HPG-axis by stimulating the synthesis and release of gonadotropin inhibitory hormone from the hypothalamus (37).

Testosterone binds with the androgen receptors (ARs) located in the brain; it can also be converted by 5‐alpha‐reductase to dihydrotestosterone (DHT) before binding to ARs. Also, it can be converted to estradiol via the aromatase pathway and bind to estrogen receptors. After binding, testosterone acts to masculinize (increase male‐typical behaviors) and defeminize (reduce female‐typical responses) behavioral development in males (46). The ARs are widely and extensively found in the medial pre-optic area (MPOA), suprachiasmatic nucleus, sexually dimorphic nucleus, and the third interstitial nucleus of the anterior hypothalamus, which are all involved in sexual behavior.

Sexual hormones regulate sexual arousal by maintaining cerebral integration between the somatic and autonomic sexual systems. Also, they assist with the ascent of sexual reflexes from the spinal cord to the cerebrum and the consequent activation of the autonomic centers leading to the organization of genital stimulation (66). Furthermore, testosterone or androgens play dominant roles in stimulating and maintaining male sexual function. Mainly, the development and growth of the penile tissue and erectile function depend on the optimal circulatory level of androgens. Supportively, androgen deficiency has been associated with penile tissue atrophy, dorsal nerve structure changes, endothelial morphology changes, reduced trabecular smooth muscle content, and alterations in the extracellular matrix (46).

Furthermore, testosterone has been shown to play key roles in sexual interest and activity. This is because loss of testosterone has been established to cause loss of libido (67, 68). The mechanism involved in libido is associated with androgens’ influence on the hypothalamic paraventricular nucleus, which is an integration center between the central and peripheral autonomic nervous systems (46).

On the other hand, prolactin is responsible for the sexual gratification that follows sexual acts (69). Prolactin is important for the refractoriness and loss of sexual drive that follows a complete sexual cycle (70). The inhibitory effect of prolactin on male function could be associated with its effect on dopamine. This is because the upregulation of prolactin receptor gene expression and prolactin-secreting lactotrophs in the anterior pituitary gland can downregulate pituitary dopamine receptor expression (71). This downregulation reduces the sensitivity of lactotrophs to dopamine’s inhibitory effects, thereby leading to hyperprolactinemia, where prolactin levels are chronically high, potentially causing various issues like infertility, and other hormonal imbalances.

On the other hand, orange peel bioactive compounds have been proven to enhance sexual functions via hormone-dependent mechanisms (15). Okesina et al. (72) established that naringin can stimulate androgen production via the HPG-axis-dependent pathway. Similarly, hesperidin has been established to enhance fertility indices by stimulating the HPG-axis to produce testosterone and suppressed prolactin secretion in a rat model of testicular dysfunction (73). Similarly, naringenin maintains hormonal balance by acting as an estrogen agonist when the level of circulatory estrogen is low and has an anti-estrogenic effect in estrogen-rich states (75). Finally, the flavonoids present in orange peel could regulate the concentration of androgens in the serum and mediate their biological effects, majorly by maintaining the HPG-axis, androgen synthesis and metabolism, androgen receptors expression, and antioxidant effects (74). Hence, orange peel bioactive compounds, especially flavonoids, regulate the HPG-axis to preserve male sexual functions and prevent psychosexual dysfunction.

#### 4.1.3 Erectile function and orange peel bioactive components

Penile erection is a complex process modulated peripherally by neurovascular, nonadrenergic, and noncholinergic mechanisms and the central nervous system (76, 77). Psychological factors and androgens regulate the erectile response and may proceed through psychogenic or reflexogenic neuronal pathways (78).

Penile erection is triggered by external erotic inputs via the five senses (Sight, Sound, Smell, Taste, and Touch), which are processed in the MPOA of the hypothalamus (79). In response to these inputs, the hypothalamus stimulates the release of monoamines and reproductive hormones such as GnRH, oxytocin, and substance P to stimulate penile erection. These reproductive hormones project to the thoracolumbar sympathetic nerve fibers at the level of T11–L2 and S2‐S4 parasympathetic nerve fibers to maintain erectile function (78).

Once external inputs stimulate the sacral parasympathetic nerves, the information is carried via the pelvic plexus to the corpora cavernosa to stimulate the release of nitric oxide (NO), the main vasoactive neurotransmitter in the penile tissue (80).

Orange peel has been established as a penile erectile function enhancement agent (12). This activity can be traced to some of the phenolic compounds present in the citrus species. Generally, flavonoids contain a C2‐3 double bond, a keto group C4, and hydroxyls at C3′ and/or C4, making them a potent fertility-enhancing agent (81). Flavonoid intake has been shown to reduce the occurrence of erectile dysfunction (82). Tangeretin is a polymethoxylated flavone, 4′,5,6,7,8-pentamethoxyflavone that has been shown to improve erectile function by modulating NO concentration (83). Similarly, naringin has been shown to act as a phosphodiesterase type 5 (PDE5) inhibitor (84). By inhibiting PDE5 activities, naringin prevented the breakdown of cGMP, thereby enhancing the activities of NO/cGMP signaling to stimulate penile erection. Also, since arginase competes with the NO/cGMP pathway for L-arginine, the inhibitory effect of naringin on arginase suggests that naringin ensures the availability of L-arginine for NO synthesis, thereby improving penile erection. Furthermore, according to Laila et al. (82), hesperidin can prevent erectile dysfunction and also enhance penile erection. Hence, phenolic compounds from orange peel can be explored as aphrodisiac agent.

## 5 Testicular functions and orange peel extract bioactive compounds

### 5.1 Spermatogenesis and Orange peel extract bioactive compounds

Spermatogenesis is a process of cell differentiation to produce fertilizing sperm, which can fuse with an ovum during fertilization to form a zygote. Spermatogenesis occurs in the testis’s Sertoli cells that isolate male germ cells from the interstitium by their tight junctions and provide nutrients for the meiosis process. (85). Orange peel bioactive compounds have been established to enhance spermatogenesis and improve sperm quality. Findings from Okesina et al. (86) and Butchi Akondi et al. (87) revealed that naringin improved sperm quality by increasing sperm count, morphology, and motility Similarly, naringenin improved total and progressive motility, viability, and membrane functionality (88). These invivo findings can be supported by those from our insilico study that reported that didymin, hesperidin, neohesperidin, naringin, narirutin, tangeretin, sinensetin, vicenin 2, and eriocitrin upregulate the activities of Arp2/3 complex via their inhibitory effect on arpin. Arp2/3 complex is responsible for maintaining acrosomal reaction, capacitation, and numerous phases of spermatogenesis, such as acrosome biogenesis, flagellum formation, and nuclear processes, such as synaptonemal complex formation (15). Also, these bioactive compounds improved the blood-testis barrier (responsible for preventing the flow of toxins while ensuring the availability of nutrients to the spermatogonial stem cells) by preserving Arp2/3 complex activities.

Also, compounds from orange peel, especially the flavonoids, are potent antioxidants and could account for their sperm quality-enhancing properties. Cellular oxidative stress occurs when the antioxidant capacity is overwhelmed by the production of oxidants such as reactive oxygen species (ROS) (89). The belief that oxidative stress is an essential factor contributing to male infertility began in the 1920s. A group of scientists from the University of Cambridge demonstrated for the first time the toxic effect of lipid peroxidation on sperm function (90). Sperm cells were first identified as susceptible to oxidative damage (91). At this junction, it is important to mention that sperm cells also generate a small amount of ROS, which is vital for capacitation and fertilization. However, excess ROS is detrimental to sperm function, and it has been shown to rapidly and irreversibly impair sperm motility in humans (92).

Citrus flavonoids are potent free radical scavengers that neutralize ROS and restore redox homeostasis in testes. For example, Okesina et al. (86) and Dong et al. (93) revealed that naringin supplementation upregulated antioxidant activities (e.g., superoxide dismutase (SOD) and catalase) and downregulated pro-oxidant expression (malondialdehyde (MDA). These effects protect testicular cells from oxidative stress and maintain sperm quality and function. Furthermore, Choi et al. (94) established the antioxidant properties of hesperidin using an *in vitro* model, while Ortiz et al. (95) reported the antioxidant activities of neohesperidin. Hence, bioactive compounds from orange peel can preserve spermatogenesis and may improve sperm quality.

### 5.2 Steroidogenesis and orange peel bioactive compounds

The multi-step process of converting cholesterol into steroid hormones is referred to as steroidogenesis. Steroidogenesis is restricted to the Leydig cells in the testis, where cholesterol is converted to testosterone. Testosterone in embryonic life is responsible for the development of male sex organs by stimulating the growth of Wolffian ducts to form epididymis, vas deferens, and seminal vesicles. Additionally, testosterone contributes to the development of genital tubercle to form the penis, scrotum, and prostate (95). At puberty, testosterone ensures brain masculinization, male sexual behavior development, spermatogenesis, external genitalia maturation, and regulation of gonadotropins. Hence, testosterone is the major male reproductive hormone, and all male sexual functions depend on its availability.

Bioactive compounds from orange peel have been shown to maintain male sexual functions by improving testosterone synthesis. For example, naringenin increased serum testosterone concentration after 10 weeks of oral administration in rats (96). Similarly, it was shown to abolish the decrease in serum testosterone and inhibin B following chemotherapeutic drug treatment (97). Similarly, hesperidin glycoside ameliorated vanadium-induced decline in testosterone synthesis (98). The ability of these bioactive compounds to stimulate testosterone synthesis could be associated with their ability to upregulate steroidogenic enzymatic activities. Hesperidin, for example, has been shown to upregulate the activities of steroidogenic acute regulatory protein, CYP11A1, CYP17A1, and 17β-Hydroxysteroid dehydrogenases (73). Hence, bioactive compounds from orange peel maintain male sexual function by modulating steroidogenic enzymatic activities.

## 6 Conclusion and future perspective

In conclusion, orange peels, frequently thrown away as trash in the citrus industry, can be recycled into valuable components that promote health. Orange peels are an inexpensive bioactive chemical source that modulates enzymes that are therapeutic targets in managing male sexual dysfunction. Therefore, this review identified different orange peel bioactive compounds and explored different methods of extraction such as supercritical fluid extraction, microwave-assisted extraction, ultrasound-assisted extraction, hot-water extraction, and solvent-based extraction. Additionally, we established the effect of these bioactive compounds on male sexual functions. As stated in this review, orange peel bioactive chemicals can improve male sexual functions by increasing the activities of the erectogenic enzymes, penile erection via the NO/cGMP signaling, libido, steroidogenesis or testosterone production, and spermatogenesis. Hence, these bioactive compounds may replace the expensive existing drugs for managing male sexual disorders. However, one of the significant challenges of exploring natural flavonoids is bioavailability. Hence, nanoformulations should be created as efficient delivery systems due to the potential therapeutic strength of orange peel bioactive. Nanocarriers such as nanoparticles and liposomes will improve the solubility, stability, and bioavailability of these agents.
